# Blood metagenomics next-generation sequencing has advantages in detecting difficult-to-cultivate pathogens, and mixed infections: results from a real-world cohort

**DOI:** 10.3389/fcimb.2023.1268281

**Published:** 2023-12-21

**Authors:** Mengjia Qian, Chang Li, Miaomiao Zhang, Yanxia Zhan, Bijun Zhu, Lingyan Wang, Qi Shen, Lei Yue, Hao Chen, Yunfeng Cheng

**Affiliations:** ^1^ Institute of Clinical Science, Zhongshan Hospital, Fudan University, Shanghai, China; ^2^ Department of Hematology, Zhongshan Hospital, Fudan University, Shanghai, China; ^3^ Department of Thoracic Surgery, Zhongshan-Xuhui Hospital, Fudan University, Shanghai, China; ^4^ Center for Tumor Diagnosis & Therapy, Jinshan Hospital, Fudan University, Shanghai, China; ^5^ Department of Hematology, Zhongshan Hospital Qingpu Branch, Fudan University, Shanghai, China

**Keywords:** metagenomics next-generation sequencing (mNGS), diagnosis, infection, blood, real-world, large cohort

## Abstract

**Background:**

Blood is a common sample source for metagenomics next-generation sequencing (mNGS) in clinical practice. In this study, we aimed to detect the diagnostic value of blood mNGS in a large real-world cohorts.

**Methods:**

Blood mNGS results of 1,046 cases were collected and analyzed along with other laboratory tests. The capabilities and accuracy of blood mNGS were compared with other conventional approaches.

**Results:**

Both the surgical department and the intensive care unit had a positive rate of over 80% in blood mNGS. The positive rate of mNGS was consistent with clinical manifestations. Among the 739 positive samples, 532 were detected as mixed infections. Compared to pathogen cultures, the negative predictive value of blood mNGS for bacteria and fungi detection was 98.9% [95%CI, 96.9%-100%], with an accuracy rate of 89.39%. When compared with polymer chain reaction, the consistency rates of blood mNGS for virus identification were remarkably high.

**Conclusions:**

Blood mNGS have significant advantages in detecting difficult-to-cultivate bacteria or fungi, viruses, and mixed infections, which benefits patients of surgery department the most. Samples other than blood are recommended for mNGS test if a specific infection is suspected. The reporting threshold and reporting criteria of blood mNGS need to be optimized.

## 1 Introduction

Since the first reported diagnosis of a patient with infection using metagenomic next-generation sequencing (mNGS) in 2014 (Wilson et al., 2014), this new pathogen detection technology has gradually been adopted and applied in clinical practice (Chapman et al., 2023). The main procedure of mNGS includes sample preprocessing, nucleic acid extraction, library preparation, sequencing, and bioinformatics analysis (Li et al., 2021).

mNGS accepts all types of samples for testing. One test can theoretically cover all pathogens including bacteria, fungi, viruses, and parasites simultaneously. Especially, mNGS performs well in identifying difficult-to-detect, rare, and co-infected pathogens (Han et al., 2019). For some patients, samples such as bronchoalveolar lavage fluid, cerebrospinal fluid, and sputum are unable to be effectively collected in clinical practice. Blood is the most convenient and common sample source for laboratory tests so that its collection, transportation, and preservation are more convenient than other sample types. In pathogenic testing, blood has been proven to be feasible in molecular diagnostic methods such as polymer chain reaction (PCR) (Chen et al., 2019; Chen et al., 2020). Recently, the efficacy of blood in pathogen detection through mNGS in various infections has been examined (Yue et al., 2021; Xu et al., 2022; Yang et al., 2022). However, the detection and diagnostic performance of blood mNGS for pathogens need to be validated in a large cohort real-worldly. Thus, the present study aimed to analyze and compare the results of peripheral blood mNGS in detecting microorganisms along with the results of conventional approaches such as pathogen culture and PCR in a large patient cohort from daily tests, to appraise the detection performance of blood mNGS, and to optimize the reporting threshold and reporting criteria of blood mNGS in real world clinical settings.

## 2 Methods

### 2.1 Patient cohort

Consecutive hospitalized patients who received blood mNGS testing at Zhongshan Hospital, Fudan University, a National Medical Center of China located in Shanghai, from January 2019 to December 2021, were enrolled to the study with their clinical records including results of laboratory tests such as pathogen cultures, PCR assay, blood routine test, C-reactive protein, procalcitonin, erythrocyte sedimentation rate, and interleukin-6 collected, compared, and analyzed. The study was approved by the institutional review board of the hospital (#B2021-694R). Written informed consent was obtained from each patient prior to enrollment.

### 2.2 mNGS assay

Peripheral blood samples were collected in a cell-free deoxyribonucleic acid (DNA) protection tube, gently mixed upside down, and placed upright at room temperature. Other samples, including sputum, bronchoalveolar lavage fluid, cerebrospinal fluid, bile, ascites, hydrothorax, pus, drainage, urine, and feces, were collected in sterile containers. The swabs were vibrated thoroughly in physiological saline, and the supernatants were collected for test. The samples were centrifuged, and the supernatants were collected for DNA extraction and purification. Extracted plasma cell-free DNA were quantified for library construction including end repair, barcoding, and PCR amplification. Qualified libraries were sequenced on the BGISEQ-50 platform.

After sequencing, the raw data were compared to human references to remove the human resource data. The remaining sequence data were aligned to the bacteria, fungi, viruses, and parasites database to calculate the final results including depth, coverage rate (CovRate), relative abundance (Re_Abu), stringent mapped read number (SMRN), etc. The results were reported according to the criteria as following (Miao et al., 2018): A) Bacteria (mycobacteria excluded), viruses, and parasites positive: the CovRate of the microbe (species level) was 10-fold greater than other microbes. B) Mycobacteria positive: SMRN≥1. C) Fungi positive: the CovRate of the microbe (species level) was 5-fold greater than other microbes. D) The background microbes, and colonized microbes were listed in separate columns of the report. Because of the high sensitivity of mNGS, the results would include common background microbes which referred to the pollution present in the laboratory environment that were determined based on daily work experience. The colonized microbes were distinguished from pathogenic microbes based on different sample types. If there were only colonized microbes or background microbes in the report, it was defined as a negative report.

### 2.3 Statistical analysis

Data were presented as number, frequencies, percentages, or mean values ± standard error whenever appropriate. Differences in categorical variables between groups were compared using the chi-squared test. Differences in continuous variables were tested for normality using Shapiro–Wilk normality test, and compared using the Student’s unpaired t-test (for parametric distribution) or the Mann–Whitney U-test (for nonparametric distribution), as appropriate. The negative predictive value (NPV) was presented with 95% confidence intervals (CIs). Data analysis was performed using the Statistical Package for Social Sciences version 16 for Windows (SPSS Inc., Chicago, IL). Two-sided p values of less than 0.05 were considered statistically significant.

## 3 Results

### 3.1 Patient characteristics

**Figure 1:**
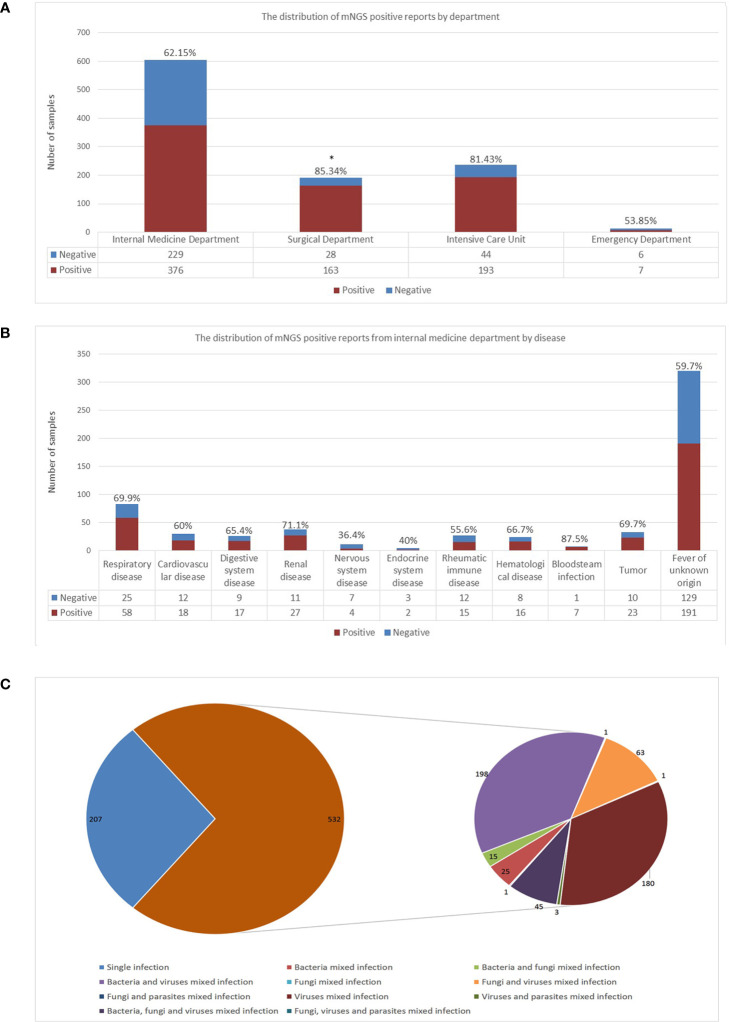
Detection performance of blood mNGS. **(A)** The distribution of mNGS positive reports in each department including internal medicine department, surgical department, intensive care unit, and emergency department. **(B)** The distribution of mNGS positive reports in each disease of internal medicine department. **(C)** Single infection and mixed infection including bacteria, fungi, and viruses detected by blood mNGS. The number in the figure represented the number of positive mNGS reports. *mNGS* metagenomics next-generation sequencing. *p<0.05, statistical difference.

The study included a total of 1,046 peripheral blood samples from hospitalized patients tested by mNGS (605 from internal medicine, 237 from the intensive care unit, 191 from the surgical department, and 13 from the emergency department), of them 739 samples were reported positive, 307 were negative (defined as reported only with colonization or background microorganisms). The distributions of positive rates by department were calculated and shown in **Figure 1A**. The surgical department had a significant higher positive rate compared to that of the internal medicine department (p=0.0007).

The distribution of positive reports of disease from the internal medicine department were further calculated and shown in **Figure 1B**. Although the number of samples tested by mNGS in patients with fever of unknown origin was the largest, the positive rate was only 59.7%. The highest positive rate was in patients with bloodstream infection. Seven out of the eight samples from patients who were suspected to have bloodstream infections were reported positive. The only one case that mNGS reported negative was a patient diagnosed extramurally with sheep brucellosis, whose blood culture was reported *sheep brucellosis* positive. However, intramurally, the patient’s anti-brucella antibody was 1:200 (positive), and the brucella nucleic acid test was negative, both results were consistent with the mNGS test.

### 3.2 Detection performance of mNGS assay

To examine the detection performance of mNGS in peripheral blood for pathogens, positive samples were classified as single infection or mixed infection. Of the 739 positive samples, 532 (71.99%) were detected as mixed infection. The most frequent pattern of mixed infection was bacteria and virus mixed infection (198 out of 532, 37.22%), followed by virus mixed infection (180 out of 532, 33.83%). Forty-five samples were reported as bacteria, fungi, and virus mixed infection (8.46%), as shown in **Figure 1C**.

### 3.3 Concordance between blood mNGS and blood culture in bacteria and fungi infection

**Figure 2:**
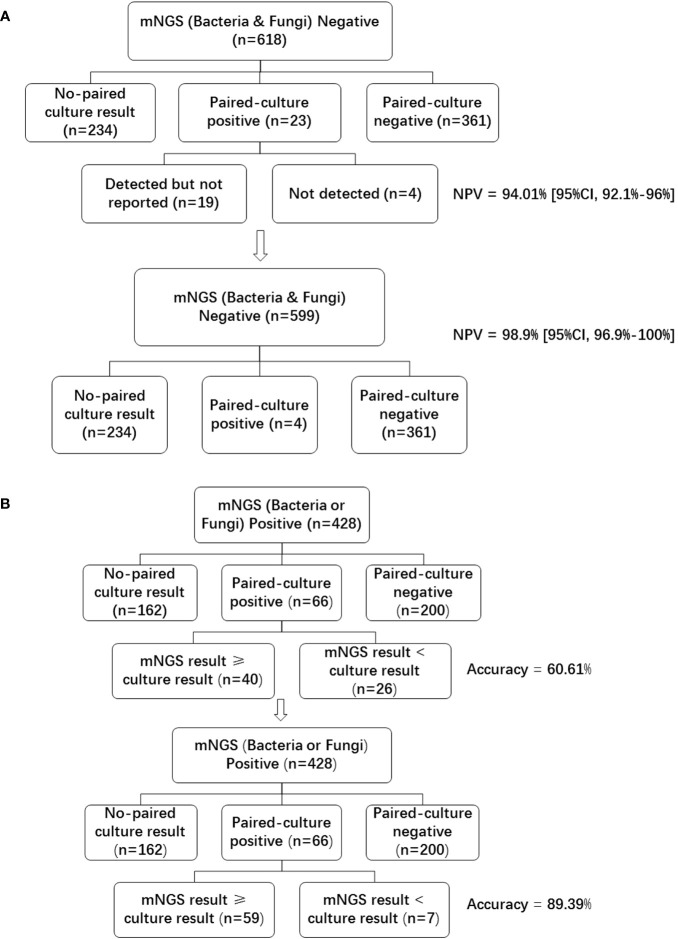
Negative predictive value and accuracy of blood mNGS compared with culture in bacteria and fungi. **(A)** Bacteria and fungi negative mNGS reports were divided into no-paired culture result, paired-culture positive, and paired-culture negative groups. Paired-culture positive cases were further divided into detected but not reported, and not detected groups. The lower graph showed the data after removing the 19 cases in the detected but not reported group. **(B)** Bacteria or fungi positive mNGS reports were divided into no-paired culture result, paired-culture positive, and paired-culture negative groups. Paired-culture positive cases were further divided into mNGS result ≥ culture result, and mNGS result < culture result groups. The lower graph showed the data after re-analyzing the detected but not reported samples (the number of mNGS result ≥ culture result group was increased to 59). *NPV* negative predictive value, *mNGS* metagenomics next-generation sequencing.

Of the 1046 samples tested with mNGS, 618 (59.08%) were bacteria and fungi negative, 428 (40.92%) were bacteria or fungi positive. Of the 618 mNGS negative samples, 361(58.41%) were paired-culture (samples for mNGS and culture were collected at the same time) negative, and 23 (3.72%) were paired-culture positive (**Figure 2A**). As compared with blood culture results, the NPV of mNGS was 361/(361 + 23) = 94.01% [95%CI, 92.1%-96%]. With the removal of 19 samples that were bacteria or fungi culture-positive, the NPV of mNGS was increased to 361/(361 + 23-19) = 98.9% [95%CI, 96.9%-100%]. These 19 cases of the 23 paired culture positive samples (**Supplementary Table 1**) were not listed in the final report due to the following reasons: listed in colonization or background; did not meet the reporting criteria; and pathogenicity was not considered.

Of the 428 bacteria or fungi positive samples, 66 (15.42%) were paired-culture positive, 200 (46.73%) were paired-culture negative. As mNGS can detect many microbes that cannot be detected by traditional methods, the false positive data of all microbes detected by mNGS could not be analyzed. Instead, accuracy was used to examaine and compare the detection performance of positive cases. In order to analyze the accuracy of mNGS, the consistent result is defined as the mNGS results contain culture results, while the inconsistent result is that the mNGS results do not comprise culture results. Under this definition, a total of 40 samples had consistent results, while 26 samples had inconsistent results (**Figure 2B**). The accuracy of mNGS when compared with culture was 40/66 = 60.61%. However, pathogens detected by culture had also been detected by the mNGS assay in 19 of the 26 samples defined as inconsistent (**Supplementary Table 2**). These pathogens were not reported due to similar reasons as mentioned above. With the removal of these 19 samples, the accuracy of mNGS was increased to (40 + 19)/66 = 89.39%.

### 3.4 Concordance between mNGS and clinical diagnosis in difficult-to-cultivate bacteria and fungi infections

For difficult-to-cultivate bacteria and fungi infections, the clinical diagnoses recorded in medical history were traced by concordance analysis of mNGS. For example, for the identification of *Pneumocystis jiroveci*, if the diagnosis of *Pneumocystis jiroveci* infection and the target therapy were recorded in the medical history, it was defined as a concordant result with mNGS. Six representative microbes that were difficult to determine through routine tests were selected and analyzed. Results showed that in patients with mNGS positive results, 78.2% *Pneumocystis jiroveci*, 54.2% *Mycobacterium tuberculosis*, 66.7% *Nocardia*, 33.3% *Mycoplasma*, 100% *Chlamydia*, and 45.5% *Rickettsia* received symptomatic treatment (**Table 1**). The remaining patients (with mNGS positive results) who did not receive symptomatic treatment were due to the following reasons: incredible result (low SMRN), patients were unwilling to receive treatment, cannot receive anti-treatment (poor liver function), sulfonamide allergy, considered to resolve the main contradiction first (critical patients), and patient death.

**Table 1:** Concordance between mNGS and clinical diagnosis in difficult-to-cultivate bacteria and fungi.

	mNGS positive sample	Symptomatic treatment case	Proportion	Reasons for not receiving symptomatic treatment
Pneumocystis jiroveci	55	43	78.20%	sulfonamide allergy/incredible result (low SMRN)/patient death
Mycobacterium tuberculosis	24	13	54.20%	critical patients should first resolve the main contradiction/patients are unwilling to receive treatment/cannot receive anti-tuberculosis treatment (poor liver function)/incredible result (low SMRN)
Nocardia	6	4	66.70%	incredible result (low SMRN)
Mycoplasma	6	2	33.30%	incredible result (low SMRN)
Chlamydia	3	3	100.00%	/
Rickettsia	11	5	45.50%	incredible result (low SMRN)

mNGS metagenomics next-generation sequencing, SMRN stringent mapped reads number at species level. /, not available.

### 3.5 Concordance between mNGS and PCR in virus infection

The PCR results of blood samples were traced and compared with blood mNGS results to examine the consistency of three common viruses including *hepatitis B virus*, *cytomegalovirus*, and *Epstein-Barr virus*, as shown in **Table 2**. The consistency rate of *Cytomegalovirus* was 100%, followed by *hepatitis B virus* (81.8%), and *Epstein-Barr virus* (80%), between the results of mNGS and PCR.

**Table 2:** Concordance between mNGS and PCR in viruses.

	PCR positive	mNGS positive	Consistency rate
**EBV**	5	4	80%
**HBV**	11	9	81.80%
**CMV**	21	21	100%

mNGS metagenomics next-generation sequencing, PCR polymer chain reaction, EBV Epstein-Barr virus, HBV hepatitis B virus, CMV Cytomegalovirus.

### 3.6 Detection performance compared between blood and other paired sample types

**Figure 3:**
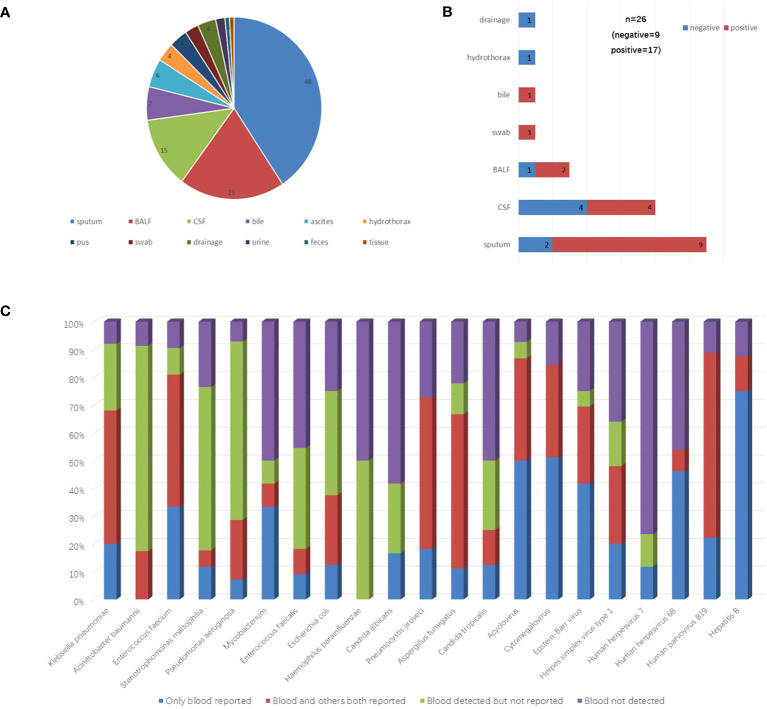
Detection performance compared between blood and other paired samples. **(A)** The distribution of other paired sample types. **(B)** The proportion of positive mNGS reports in other sample types correlated with negative blood mNGS results. **(C)** The detection performance of microbes which the total detected case >5 were shown in the figure. These microbes were divided into four groups including only blood reported, blood and others both reported, blood detected but not reported, and blood not detected. *BALF* bronchoalveolar lavage fluid, *CSF* cerebrospinal fluid.

To examine the potential of peripheral blood in replacing other sample types for mNGS assay, the mNGS results of 118 paired with other sample types from 110 cases were analyzed. The distribution of these sample types was shown in **Figure 3A**. Among them, 48 (40.68%) were sputum, 23 (19.49%) were bronchoalveolar lavage fluid.

Of these 118 samples, 26 were blood mNGS negative, 92 were positive. Specifically, among the 26 mNGS negative samples, 17 of their paired samples were positive, 9 of their paired samples were negative (**Figure 3B**). A total of 36 pathogens reported positive in the 17 paired positive samples that were not reported in blood mNGS were reanalyzed. Among them, 17 (47.2%) pathogens were actually detected but not listed in the blood report according to the reporting criteria (**Supplementary Table 3**).

The results of 92 samples of other types correlated with positive blood mNGS results. Among them, 22 had consistent results with blood. Seventy samples had inconsistent results with blood. A total of 220 microbes were reported in these 70 samples but not listed in the blood reports, however, 88 (40%) microbes were actually detected (**Supplementary Table 4**).

To further evaluate the detection performance of blood mNGS, all detected microbes in these 118 samples were analyzed. The detection performances of microbes including bacteria, fungi, and viruses (total detected cases >5) were shown in **Figure 3C**.

### 3.7 Comparison of blood mNGS with blood tests

**Figure 4:**
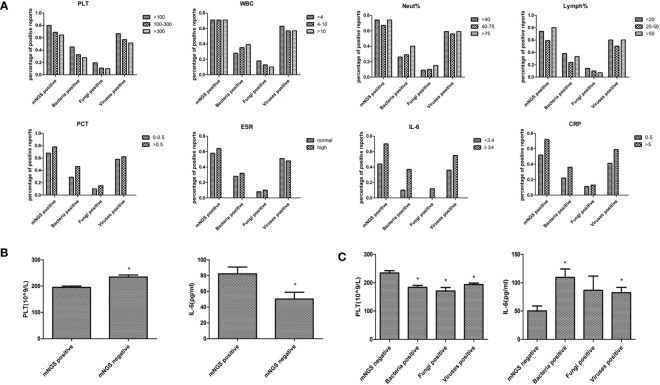
Comparison of blood mNGS with blood routine test and inflammatory indexes. **(A)** Blood routine test results including PLT(10^9^/L), WBC (10^9^/L), Neut (%), Lymph (%), and inflammatory indexes results including PCT (ng/ml), ESR (mm/H), IL-6 (pg/ml), and CRP (mg/L) were collected to compare the number of positive blood mNGS reports with the clinical manifestations. **(B)** The significant differences in PLT number and IL-6 concentration between the mNGS positive and negative groups. **(C)** The changes of PLT number and IL-6 concentration in mNGS negative, bacteria positive, fungi positive, and viruses positive groups. *PLT* platelet, *WBC* leukocyte, *Neut* neutrophil, *Lymph* lymphocyte, *PCT* procalcitonin, *ESR* erythrocyte sedimentation rate, *IL-6* interleukin-6 (IL-6), *CRP* creactive protein, *mNGS* metagenomics next-generation sequencing. *p<0.05, statistical difference.

Blood routine test results, including platelet, leukocyte, neutrophil, lymphocyte, and inflammatory indexes results, including procalcitonin, erythrocyte sedimentation rate, interleukin-6, and C-reactive protein were collected to compare the blood mNGS results with the clinical manifestations (**Figure 4A**). The percentage of positive mNGS reports of either bacteria, fungi, or viruses were all increased with elevated levels of each inflammatory index. However, the percentage of positive mNGS reports were decreased with the increase of platelet counting. The percentage of positive mNGS reports of bacteria were increased with the elevation of white blood cell counting and neutrophil percentages. Similarly, the percentages of blood mNGS positive reports were consistent with the trend of the changes of lymphocyte percentage. Significant differences were found in the platelet number and IL-6 concentration between the mNGS positive and mNGS negative groups (**Figure 4B**). Moreover, the platelet numbers in either bacteria, fungi, or virus positive groups were all significantly lower than in the mNGS negative group (**Figure 4C**).

### 3.8 Impact of laboratory calibration on blood mNGS results

**Figure 5:**
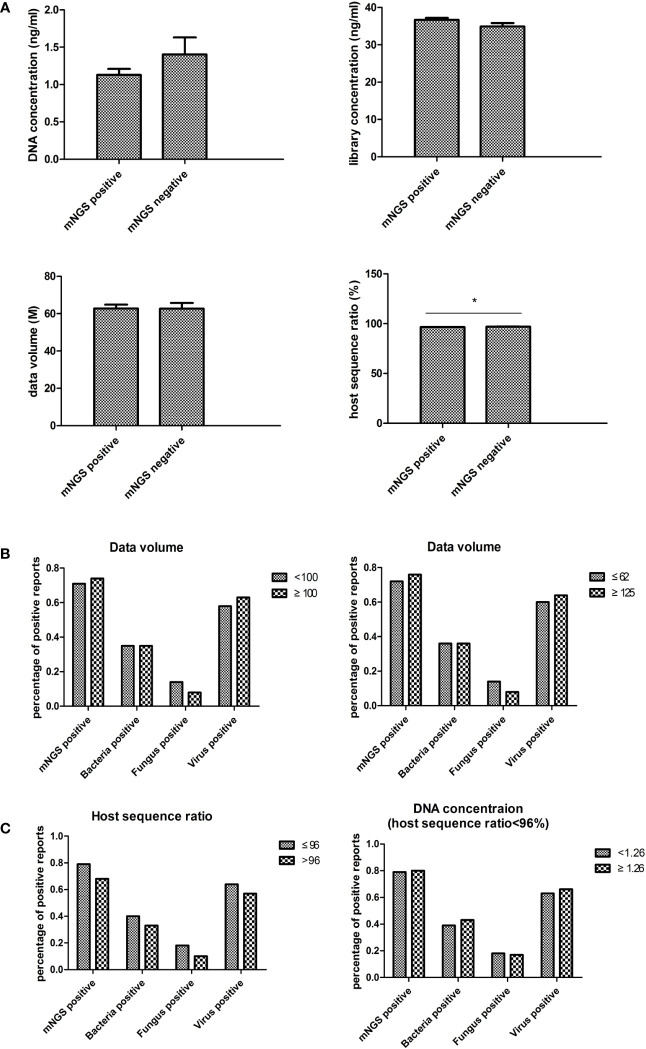
Laboratory impact on blood mNGS results. **(A)** The experimental data including DNA concentration, library concentration, data volume, and host sequence ratio were collected to evaluate the laboratory impact on blood mNGS results. **(B)** The correlation between positive mNGS reports with data volume. **(C)** The correlation between host sequence ratio and DNA concentration. *DNA* deoxyribonucleic acid, *mNGS* metagenomics next-generation sequencing. *p<0.05, statistical difference.

The mNGS experimental data, including DNA concentration, library concentration, data volume, and host sequence ratio, were collected to evaluate the impact of laboratory settings on blood mNGS results (**Figure 5A**). Only the host sequence ratio showed a difference between the mNGS positive and negative groups.

In order to validate the impact of data volume, a data volume of 100M was used as the cutoff value for comparison. No significant differences were found between groups. Subsequently, the cutoff value was set to 62M (the average value of this batch of samples) and 125M (twice the average value), yet still no significant differences were found (**Figure 5B**).

To examine the necessity of tracing DNA concentration in low host sequence ratio samples, the results between samples of less than 96% host sequence ratio (the average value of this batch of data) and greater than 96% host sequence ratio were compared. The results showed that the percentage of positive reports had an inverse trend compared to the host sequence ratio (**Figure 5C**). Then the correlation between DNA concentration and the percentage of positive reports in the group of less than 96% host sequence ratio was analyzed. However, no differences were found in each of the groups (**Figure 4C**).

## 4 Discussion

Peripheral blood mNGS assay has recently been adopted as a supplementary approach to detect infectious microorganisms (Deng et al., 2023). Yet no large sample size studies have comprehensively apprised the clinical values of this approach. In the current study, 1,046 peripheral blood mNGS reported from a large real-world cohort were compared and analyzed with conventional laboratory tests. The overall positive rate of blood mNGS results was 70.7% in our institution. The surgical department had the highest positive rate (85.34%), followed by the intensive care unit (81.43%). Nearly 60% of the mNGS samples were from the internal medicine department, with an overall positive rate of 62.15%. Fever of unknown origin is still a challenge for clinicians which urgently need new methods to assist in diagnosis (Wright and Auwaerter, 2020). Among them, the positive rate of fever of unknown origin was 59.7%, suggesting that the blood mNGS assay alone could not completely address this challengeable clinical problem, and that integration with other approaches is apparently necessary to achieve diagnostic precision (Dong et al., 2022; Zhang et al., 2022). The positive rate of suspected bloodstream infection was 87.5%, which was consistent with another report (Jing et al., 2021). The significant difference in positive rate between the surgical department and the internal medicine department may be due to the difference in the timing of testing. The surgical departments pursues timeliness and often has mNGS tests in the early stage of infection, while the internal medicine departments often has mNGS tests when pathogens cannot be detected through other conventional tests. The overall positive rate of the emergency department (53.85%) was the lowest as compared with other departments, suggesting that blood mNGS might not be blindly recommended for patients under emergency circumstances.

The predominant advantage of mNGS is that this technology could simultaneously detect all pathogens, including bacteria, fungi, viruses, and parasites, in one test (Qian et al., 2022). Several studies have confirmed this advantage of mNGS, and the timely use of mNGS was recommended when mixed infection was suspected (Qian et al., 2020; Zhao et al., 2021; Chen et al., 2022). In the present study, 71.99% of the blood samples were detected mixed infection by mNGS. The most common pattern was bacteria and virus mixed infection (198/532), followed by viruses mixed infection (180/532), demonstrating the advantage of blood samples in virus detection. Notably, 45 samples were detected as bacteria, fungi, and virus mixed infection, which underscored the capabilities of blood mNGS in mixed infection detection.

The potential and values of blood mNGS in pathogen detection were compared with conventional approaches for bacteria, fungi, and viruses, respectively, to apprise the application of blood mNGS in real-world clinical practice. For most bacteria and fungi, when compared with culture, the NPV of blood mNGS was 98.9% [95%CI, 96.9%-100%], with an accuracy rate of 89.39%, if samples in that pathogen were detected but not reported were included (**Figure 2**). The reasons for pathogens that were culture-positive but not listed in the mNGS reports were, firstly, listed in the background or colonized microorganism list, such as *Staphylococcus epidermidis* is one of the most common bacteria of the human skin microbiota (Goncalves et al., 2022; Wang et al., 2023). If detected in blood mNGS, it is usually listed in the list of colonized microorganisms. Second, the pathogen detected did not meet the reporting criteria. For example, in sample 743, the culture result was *Acinetobacter baumannii* positive, however the CovRate was low in mNGS detection, and it was excluded in the mNGS report per the current reporting criteria of our institution. Blood mNGS has the advantage of time conserving over conventional culture, as the entire process of mNGS only takes 24-48 hours (Indelli et al., 2021) (24 hours in our institution),with the high accuracy and NPV demonstrated in the study, mNGS is strongly supported as an alternative routine method for blood culture.

For difficult-to-cultivate bacteria or fungi infections, six typical pathogens which were difficult to diagnose in clinical practice were selected for consistency evaluation based on clinical diagnosis and medication. A total of 55 cases with *Pneumocystis jiroveci* were positive in the blood mNGS test, of which 43 underwent targeted treatment. The result of mNGS would be very valuable when the clinical diagnosis of this bacterium infection was mainly based on clinical symptoms and radiographic findings (Bateman et al., 2020). For *Mycobacterium tuberculosis*, the commonly used methods to diagnose are microscopy, radiography, culture, and molecular assays (Acharya et al., 2020). We found that 13 out of 24 cases with mNGS positive results received symptomatic treatment, implying that mNGS would provide a new option for *Mycobacterium tuberculosis* diagnosis. The diagnosis of *Nocardia* infection is limited worldwide because of the difficulties in its isolation and incubation (Ding et al., 2021). However, in the current study, 6 cases were detected *Nocardia* positive, supporting mNGS as a putative tool for *Nocardia* identification. Given superior sensitivity detected of difficult-to-cultivate bacteria and fungi, the blood mNGS test is highly recommended to assist diagnosis when these pathogens were clinically suspected.

The concordance between blood mNGS and PCR in virus detection including *Epstein-Barr virus*, *hepatitis B virus*, and *cytomegalovirus* was also examined in the present study. The consistency rates of mNGS with PCR results uniformly reached over 80% for these three representative viruses, indicating that mNGS has a higher consistency compared to conventional methods. To our best knowledge, there is no other study that has evaluated the result consistency of these three viruses between blood mNGS and PCR were reported.

Furthermore, a total of 118 paired samples including sputum, bronchoalveolar lavage fluid, cerebrospinal fluid, and others were analyzed to evaluate the possibility of using peripheral blood samples to replace other sample types in the mNGS test. We found that when pathogens were detected in bronchoalveolar lavage fluid and sputum samples, a large portion of the paired blood mNGS was negative, suggesting that when respiratory infections are suspected, bronchoalveolar lavage fluid and sputum are the preferred sample sources for mNGS test if they are feasible to collect. Through the deepened analysis of the main detected pathogens from both blood and other samples, blood samples had significant advantages in virus detection when compared to other sample types, which was consistent with the findings above (**Figure 1C**). Additionally, by comparing with other samples, bacteria such as *Acinetobacter baumannii*, *Stenotrophomonas maltophilia*, and *Pseudomonas aeruginosa*, the portion of “blood detected but not reported” reached to 73.9%, 58.8%, and 64.3%, respectively (**Figure 3C**). Therefore, it might be necessary to tailor the blood reporting threshold appropriately.

Blood routine tests and infection indexes were collected on the day of mNGS examination to determine their impact on the positive rate of blood mNGS. The positive rate of mNGS was consistent with the changes in each of the infectious indexes, suggesting that the results of mNGS could indeed provide an auxiliary diagnosis. Notably, platelet number was significantly correlated with the positive rate of either bacteria, fungi, or viruses in mNGS detection, suggesting that more attention should be paid to the role of platelets in infection. There were no significant differences between the mNGS negative group and the mNGS positive group in experimental variables such as DNA concentration, library concentration, and data volume. There were no differences found even when data volumes between 62M and 125M were compared. As such, empirically increasing data volume might help detect more pathogens in other sample types, however, this maneuver is not applicable to blood mNGS from our real-world data. Only in terms of host sequence ratio, a significant difference was found between the negative and positive groups. It is empirically believed that it is necessary to trace the DNA concentration to confirm the reliability of the result if the host sequence ratio is low. However, analysis of real-world data suggested that this approach was unnecessary.

There are several limitations that need to be acknowledged. Firstly, only DNA was extracted for mNGS test, the results of viruses only including DNA viruses, Ribonucleic Acid (RNA) viruses were not enrolled in the study. Whether blood mNGS have advantages in the detection of RNA viruses needs to be further explored. Secondly, portions of the enrolled samples were collected after antibiotics therapy, it was not possible to exclude the impact of antibiotics on the detection performance of blood mNGS.

## 5 Conclusions

The current study demonstrated the significant advantages of blood mNGS in detecting difficult-to-cultivate bacteria or fungi, viruses, and mixed infections. It is recommended for clinicians to perform a blood mNGS tests for patients with suspected infections, especially at the surgery department. If respiratory infection is suspected, other types of samples are more recommended for mNGS other than peripheral blood. Blindly increasing data volume would not improve the positive rate of blood mNGS. The laboratory should appropriately tailor the reporting threshold and optimize the suitable reporting criteria for blood samples.

## Data availability statement

The original contributions presented in the study are included in the article/**Supplementary Material**. Further inquiries can be directed to the corresponding author.

## Author contributions

MQ: Conceptualization, Data curation, Formal analysis, Investigation, Methodology, Resources, Writing – original draft, Writing – review & editing. CL: Data curation, Methodology, Resources, Software, Writing – review & editing. MZ: Data curation, Formal analysis, Writing – review & editing. YZ: Data curation, Formal analysis, Methodology, Software, Writing – review & editing. BZ: Data curation, Methodology, Writing – review & editing. LW: Writing – review & editing. QS: Data curation, Methodology, Writing – review & editing. LY: Conceptualization, Data curation, Methodology, Writing – review & editing. HC: Conceptualization, Resources, Supervision, Writing – review & editing, Writing – original draft. YC: Conceptualization, Funding acquisition, Investigation, Methodology, Resources, Supervision, Writing – original draft, Writing – review & editing.
