# Supplementary material for: Blood metagenomics next-generation sequencing has advantages in detecting difficult-to-cultivate pathogens, and mixed infections: results from a real-world cohort
Source: Front Cell Infect Microbiol. 2023 Dec 21;13:1268281. doi: 10.3389/fcimb.2023.1268281 (PMC10768086; doi:10.3389/fcimb.2023.1268281)
Supplement: Supplementary file 1 [file Table_1.docx]

|  | **Culture result** | **mNGS result** | | | | | | | | |
| --- | --- | --- | --- | --- | --- | --- | --- | --- | --- | --- |
| **Sample** | **Culture result** | **CovRate** | **Depth** | **Re_Abu** | **Genus_Re_Abu** | | **SMRN** | | **SMRNG** | |
| 117 | Raoultella ornithinolytica | 0.0013 | 1 | 0.1 | 0.1 | | 1 | | 1 | |
| 152 | Staphylococcus epidermidis | 0.3764 | 1 | 2.62 | 12.31 | | 182 | | 1118 | |
| 158 | Staphylococcus epidermidis | 0.0072 | 1 | 10.46 | 17.96 | | 4 | | 7 | |
| 165 | Acinetobacter johnsonii | 32.09 | 1.45 | 79.91 | 96.17 | | 48023 | | 63803 | |
| 184 | Enterococcus faecium | 0.0218 | 1 | 0.16 | 0.22 | | 14 | | 20 | |
| 187 | Acinetobacter baumannii | 0.001 | 1 | 0.01 | 2.12 | | 1 | | 136 | |
|  | Klebsiella pneumoniae | 0 | 0 | 0 | 0 | | 0 | | 0 | |
| 191 | Candida parapsilosis | 0.0026 | 1 | 1.63 | 1.63 | | 4 | | 5 | |
| 194 | Staphylococcus hominis | 0.4586 | 1 | 0.92 | 4.49 | | 254 | | 1436 | |
| 197 | Staphylococcus | / | / | / | 2.46 | | / | | 46 | |
| 266 | Staphylococcus haemolyticus | 0.4053 | 1 | 8.26 | 43.19 | | 231 | | 1128 | |
| 379 | Pseudomonas aeruginosa | 0.0005 | 1 | 0.07 | 0.94 | | 0 | | 2 | |
| 412 | Stenotrophomonas maltophilia | 0 | 0 | 0 | 0 | | 0 | | 0 | |
| 466 | Staphylococcus | / | / | / | 1.64 | | / | | 117 | |
| 576 | Staphylococcus haemolyticus | 0 | 0 | 0 | 0 | | 0 | | 0 | |
| 743 | Acinetobacter baumannii | 0.0024 | 1 | 0.11 | 0.59 | | 1 | | 4 | |
| 766 | Bacillus siamensis | 0 | 0 | 0 | 0 | | 0 | | 0 | |
| 782 | Staphylococcus | / | / | / | 2.13 | | / | | 152 | |
| 785 | Streptococcus gallolyticus | 0.0402 | 1 | 0.53 | 1.7 | | 6 | | 40 | |
| 803 | Staphylococcus epidermidis | 0.2592 | 1 | 1 | 3.74 | | 110 | | 373 | |
| 832 | Staphylococcus | / | / | / | 0.45 | / | | 5 | |  |
| 908 | Staphylococcus | / | / | / | 2.85 | | / | | 2 | |
| 1013 | Staphylococcus epidermidis | 0.0019 | 1 | 0.43 | 2.27 | | 1 | | 4 | |
| 1024 | Stenotrophomonas maltophilia | 0.0019 | 1 | 0.06 | 0.15 | | 3 | | 3 | |
|  | Staphylococcus | / | / | / | 0.25 | | / | | 8 | |

**Supplementary material captions**

**Supplementary table 1**

List of 23 paired-culture positive samples.

**Supplementary table 2**

List of 26 samples defined as inconsistent results.

|  | **Culture result** | **mNGS result** | | | | | |
| --- | --- | --- | --- | --- | --- | --- | --- |
| **Sample** | **Culture result** | **CovRate** | **Depth** | **Re_Abu** | **Genus_Re_Abu** | **SMRN** | **SMRNG** |
| 50 | Citrobacter freundii | 0 | 0 | 0 | 0 | 0 | 0 |
| 89 | Staphylococcus epidermidis | 0.0717 | 1 | 0.81 | 14.86 | 36 | 694 |
| 126 | Acinetobacter johnsonii | 0.0458 | 1 | 1.5 | 2.2 | 37 | 59 |
| 247 | Proteus mirabilis | 0.0028 | 1 | 0.11 | 0.11 | 3 | 3 |
| 260 | Staphylococcus haemolyticus | 0.099 | 1 | 1.02 | 28 | 53 | 1458 |
| 269 | Enterobacter cloacae | 0.0031 | 1 | 0.01 | 0.11 | 0 | 18 |
|  | Candida albicans | 0.0156 | 1.01 | 8.97 | 9.75 | 92 | 100 |
| 302 | Enterobacter cloacae | 0.0134 | 1 | 0.91 | 28.98 | 0 | 452 |
| 367 | Staphylococcus warneri | 0 | 0 | 0 | 0 | 0 | 0 |
| 373 | Candida parapsilosis | 0 | 0 | 0 | 0 | 0 | 0 |
| 382 | Staphylococcus epidermidis | 0.0015 | 1 | 1.09 | 8.83 | 1 | 7 |
| 385 | Staphylococcus sciuri | 0.008 | 1 | 3.16 | 27.91 | 4 | 61 |
|  | Enterococcus avium | 0.0073 | 1 | 1.89 | 10.18 | 7 | 29 |
| 415 | Stenotrophomonas maltophilia | 0.0029 | 1 | 0.06 | 0.08 | 3 | 4 |
| 420 | Staphylococcus capitis | 0 | 0 | 0 | 0 | 0 | 0 |
| 442 | Staphylococcus haemolyticus | 0 | 0 | 0 | 0 | 0 | 0 |
| 502 | Staphylococcus capitis | 0.0115 | 1 | 0.23 | 1.04 | 2 | 21 |
| 509 | Klebsiella aerogenes | 0.0008 | 1 | 0.0056 | 0.0107 | 1 | 1 |
|  | Staphylococcus | / | / | / | 1.76 | / | 402 |
| 523 | Staphylococcus | / | / | / | 0.81 | / | 30 |
| 537 | Staphylococcus hominis | 0.0163 | 1 | 0.37 | 2.51 | 9 | 65 |
| 548 | Micrococcus luteus | 0.0019 | 1 | 0.02 | 0.09 | 1 | 3 |
| 586 | Staphylococcus epidermidis | 0.0101 | 1 | 0.15 | 1.16 | 4 | 37 |
| 661 | Staphylococcus haemolyticus | 0.0422 | 1 | 1.06 | 2.62 | 21 | 51 |
| 673 | Raoultella ornithinolytica | 0.0015 | 1 | 0.07 | 0.19 | 2 | 5 |
| 735 | Klebsiella pneumoniae | 0.2428 | 1 | 9.32 | 10.87 | 93 | 257 |
| 767 | Candida albicans | 0 | 0 | 0 | 0 | 0 | 0 |
| 847 | Enterococcus faecalis | 0.005 | 1 | 0.04 | 0.14 | 1 | 7 |
| 901 | Klebsiella pneumoniae | 0 | 0 | 0 | 0 | 0 | 0 |

**Supplementary table 3**

A total of 36 pathogens reported positive in the 17 paired positive samples that were not reported in blood mNGS were listed.

| **mNGS result of other sample type** | | | **mNGS result of blood** | | | | | |
| --- | --- | --- | --- | --- | --- | --- | --- | --- |
| **Sample** | **Type** | **Result** | **CovRate** | **Depth** | **Re_Abu** | **Genus_Re_Abu** | **SMRN** | **SMRNG** |
| 10 | Sputum | Human_herpesvirus_4 | 0 | 0 | 0 | 0 | 0 | 0 |
|  |  | Human_herpesvirus_4_type_2 | 0 | 0 | 0 | 0 | 0 | 0 |
| 40 | CSF | Streptococcus_pneumoniae | 0.0117 | 1 | 0.03 | 0.36 | 0 | 56 |
| 243 | Sputum | Acinetobacter_baumannii | 0.2276 | 1 | 2.68 | 10.14 | 136 | 727 |
|  |  | Klebsiella_pneumoniae | 0.0046 | 1 | 0.05 | 0.09 | 1 | 12 |
|  |  | Human_betaherpesvirus_7 | 0 | 0 | 0 | 0 | 0 | 0 |
| 303 | Biliarya | Enterococcus_faecalis | 0 | 0 | 0 | 0 | 0 | 0 |
|  |  | Enterococcus_raffinosus | 0 | 0 | 0 | 0 | 0 | 0 |
|  |  | Human_betaherpesvirus_5 | 0 | 0 | 0 | 0 | 0 | 0 |
| 441 | Sputum | Lautropia_mirabilis | 0.1394 | 1 | 4.72 | 4.72 | 76 | 76 |
|  |  | Stenotrophomonas_maltophilia | 0.001 | 1 | 0.03 | 0.23 | 1 | 2 |
|  |  | Tannerella_forsythia | 0 | 0 | 0 | 0 | 0 | 0 |
|  |  | Acinetobacter_baumannii | 0.0012 | 1 | 0.04 | 4.04 | 0 | 2 |
| 475 | Sputum | Klebsiella_pneumoniae | 0 | 0 | 0 | 0 | 0 | 0 |
|  |  | Mycoplasma_hominis | 0 | 0 | 0 | 0 | 0 | 0 |
| 636 | Swab | Pneumocystis_jirovecii | 0 | 0 | 0 | 0 | 0 | 0 |
|  |  | Human_herpesvirus_4 | 0 | 0 | 0 | 0 | 0 | 0 |
| 715 | CSF | Mycobacterium_tuberculosis_complex_group | 0 | 0 | 0 | 0 | 0 | 0 |
| 731 | Sputum | Acinetobacter_baumannii | 0.0122 | 1 | 0.29 | 1.71 | 8 | 43 |
|  |  | Stenotrophomonas_maltophilia | 0.0022 | 1 | 0.05 | 0.05 | 1 | 1 |
| 732 | Sputum | Acinetobacter_baumannii | 0.0047 | 1 | 1.24 | 1.96 | 2 | 4 |
|  |  | Stenotrophomonas_maltophilia | 0.0011 | 1 | 0.28 | 0.28 | 1 | 1 |
|  |  | Human_herpesvirus_1 | 0.0328 | 1 | 0.42 | / | 5 | / |
| 754 | CSF | Mycobacterium_tuberculosis_complex_group | 0 | 0 | 0 | 0 | 0 | 0 |
| 766 | Sputum | Enterococcus_faecium | 0 | 0 | 0 | 0 | 0 | 0 |
|  |  | Haemophilus_parainfluenzae | 0.0024 | 1 | 0.21 | 0.21 | 0 | 0 |
|  |  | Human_herpesvirus_4 | 0 | 0 | 0 | 0 | 0 | 0 |
| 781 | Sputum | Acinetobacter_baumannii | 0.583 | 1 | 51.01 | 58.32 | 260 | 439 |
|  |  | Stenotrophomonas_maltophilia | 0 | 0 | 0 | 0 | 0 | 0 |
|  |  | Human_herpesvirus_1 | 0.4019 | 1 | 5.79 | / | 19 | / |
| 794 | BALF | Mycobacterium_tuberculosis_complex_group | 0 | 0 | 0 | 0 | 0 | 0 |
| 796 | Sputum | Klebsiella_pneumoniae | 0.1631 | 1 | 2.24 | 2.83 | 71 | 169 |
| 877 | CSF | Brevundimonas_vesicularis | 0.0489 | 1 | 1.26 | 1.6 | 19 | 26 |
|  |  | Human_herpesvirus_4 | 0 | 0 | 0 | 0 | 0 | 0 |
| 1011 | BALF | Human_herpesvirus_1 | 0 | 0 | 0 | 0 | 0 | 0 |
|  |  | Human_herpesvirus_7 | 0.9747 | 1 | 93.9 | / | 1 | / |

**Supplementary table 4**

A total of 70 paired-samples had inconsistent results with blood were listed.

| **mNGS result of other sample type** | | | **mNGS result of blood** | | | | | |
| --- | --- | --- | --- | --- | --- | --- | --- | --- |
| **Sample** | **Type** | **Result** | **CovRate** | **Depth** | **Re_Abu** | **Genus_Re_Abu** | **SMRN** | **SMRNG** |
| 57 | Sputum | Torque_teno_virus | 0 | 0 | 0 | 0 | 0 | 0 |
| 74 | Sputum | Candida_orthopsilosis | 0 | 0 | 0 | 0 | 0 | 0 |
|  |  | Human_alphaherpesvirus_1 | 0 | 0 | 0 | 0 | 0 | 0 |
| 159 | BALF | Human_alphaherpesvirus_1 | 0.1187 | 1 | 0.72 | / | 6 | / |
| 163 | Swab | Acinetobacter_baumannii | 0.0192 | 1 | 0.14 | 3.17 | 11 | 339 |
|  |  | Human_alphaherpesvirus_1 | 0 | 0 | 0 | 0 | 0 | 0 |
| 170 | Sputum | Stenotrophomonas_maltophilia | 0.0168 | 1 | 0.19 | 0.24 | 6 | 7 |
|  |  | Ureaplasma_parvum | 0 | 0 | 0 | 0 | 0 | 0 |
|  |  | Acinetobacter_baumannii | 0.0102 | 1 | 0.16 | 5.3 | 6 | 364 |
| 170 | Urine | Candida_albicans | 0 | 0 | 0 | 0 | 0 | 0 |
| 196 | Swab | Pseudomonas_aeruginosa | 0.0054 | 1 | 0.05 | 0.8 | 3 | 87 |
|  |  | Enterococcus_faecalis | 0 | 0 | 0 | 0 | 0 | 0 |
|  |  | Helcococcus_kunzii | 0 | 0 | 0 | 0 | 0 | 0 |
|  |  | Facklamia_languida | 0 | 0 | 0 | 0 | 0 | 0 |
| 212 | Sputum | Enterococcus_faecium | 0.0859 | 1.01 | 0.67 | 1.18 | 56 | 106 |
|  |  | Candida_albicans | 0.0033 | 1 | 0.05 | 0.07 | 20 | 23 |
|  |  | Human_betaherpesvirus_7 | 0 | 0 | 0 | 0 | 0 | 0 |
| 215 | BALF | Acinetobacter_baumannii | 0.0648 | 1 | 0.45 | 3.18 | 41 | 396 |
| 217 | Sputum | Enterococcus_faecium | 0 | 0 | 0 | 0 | 0 | 0 |
| 220 | Sputum | Pseudomonas_aeruginosa | 0.0006 | 1 | 0.05 | 0.9 | 0 | 6 |
|  |  | Enterococcus_faecalis | 0.0013 | 1 | 0.12 | 0.48 | 1 | 2 |
|  |  | Human_betaherpesvirus_7 | 0 | 0 | 0 | 0 | 0 | 0 |
|  |  |  |  |  |  |  |  |  |
| 239 | BALF | Acinetobacter_baumannii | 0.0336 | 1 | 0.11 | 0.69 | 26 | 168 |
|  |  | Pseudomonas_aeruginosa | 0.0068 | 1 | 0.02 | 0.45 | 2 | 38 |
|  |  | Staphylococcus_aureus | 0.0052 | 1 | 0.01 | 0.16 | 1 | 33 |
|  |  | Aeromonas_dhakensis | 0.0009 | 1 | 0 | 0.04 | 0 | 7 |
|  |  | Human_betaherpesvirus_6B | 0 | 0 | 0 | 0 | 0 | 0 |
|  |  | Torque_teno_virus_27 | 0 | 0 | 0 | 0 | 0 | 0 |
|  |  | Torque_teno_virus_28 | 6.26 | 1.11 | 7.23 | / | 2 | / |
| 260 | Sputum | Stenotrophomonas_maltophilia | 0.0009 | 1 | 0.02 | 0.12 | 1 | 3 |
|  |  | Chryseobacterium_indologenes | 0.0008 | 1 | 0.01 | 6.3 | 0 | 324 |
|  |  | Human_alphaherpesvirus_2 | 0.2764 | 1 | 0.95 | / | 1 | / |
| 271 | Sputum | Candida_albicans | 0.0002 | 1 | 0.01 | 0.01 | 1 | 1 |
| 287 | BALF | Stenotrophomonas_maltophilia | 0.0081 | 1 | 0.56 | 0.88 | 2 | 4 |
|  |  | Candida_tropicalis | 0.0003 | 1 | 0.41 | 0.41 | 1 | 1 |
|  |  | Candida_parapsilosis | 0 | 0 | 0 | 0 | 0 | 0 |
|  |  | Candida_orthopsilosis | 0 | 0 | 0 | 0 | 0 | 0 |
|  |  | Candida_albicans | 0 | 0 | 0 | 0 | 0 | 0 |
| 287 | Ascitic fluid | Rothia_mucilaginosa | 0 | 0 | 0 | 0 | 0 | 0 |
|  |  | Actinomyces_graevenitzii | 0 | 0 | 0 | 0 | 0 | 0 |
|  |  |  |  |  |  |  |  |  |
| 291 | Sputum | Klebsiella_aerogenes | 0.0007 | 1 | 0.01 | 0.03 | 0 | 4 |
|  |  | Klebsiella_pneumoniae | 0.0008 | 1 | 0.01 | 0.03 | 1 | 4 |
|  |  | Kluyvera_cryocrescens | 0 | 0 | 0 | 0 | 0 | 0 |
|  |  | Acinetobacter_baumannii | 0.0103 | 1 | 0.11 | 6.35 | 5 | 476 |
|  |  | Enterobacter_cloacae | 0 | 0 | 0 | 0 | 0 | 0 |
|  |  | Enterococcus_faecalis | 0 | 0 | 0 | 0 | 0 | 0 |
|  |  | Human_herpesvirus_4 | 0 | 0 | 0 | 0 | 0 | 0 |
| 302 | Bile | Enterobacter_cloacae | 0.0134 | 1 | 0.91 | 28.98 | 0 | 452 |
|  |  | Citrobacter_werkmanii | 0.0016 | 1 | 0.08 | 0.46 | 1 | 2 |
|  |  | Kluyvera_ascorbata | 0 | 0 | 0 | 0 | 0 | 0 |
|  |  | Escherichia_coli | 0 | 0 | 0 | 0 | 0 | 0 |
|  |  | Klebsiella_aerogenes | 0.0014 | 1 | 0.07 | 0.2 | 0 | 2 |
|  |  | Serratia_marcescens | 0 | 0 | 0 | 0 | 0 | 0 |
|  |  | Human_betaherpesvirus_5 | 0 | 0 | 0 | 0 | 0 | 0 |
|  |  | Hepatitis_B_virus_subtype_adr | 5.35 | 1 | 24.64 | / | 0 | / |
| 322 | Bile | Human_betaherpesvirus_6B | 0 | 0 | 0 | 0 | 0 | 0 |
| 323 | Drainage | Proteus_mirabilis | 0.0039 | 1 | 2.41 | 2.41 | 3 | 3 |
| 462 | Sputum | Pseudomonas_aeruginosa | 0.0107 | 1.08 | 0.31 | 0.76 | 5 | 9 |
|  |  | Lactobacillus_paracasei | 0 | 0 | 0 | 0 | 0 | 0 |
|  |  | Citrobacter_freundii | 0 | 0 | 0 | 0 | 0 | 0 |
| 478 | Sputum | Human_herpesvirus_4 | 0 | 0 | 0 | 0 | 0 | 0 |
| 480 | Sputum | Human_betaherpesvirus_7 | 0 | 0 | 0 | 0 | 0 | 0 |
|  |  |  |  |  |  |  |  |  |
| 507 | Sputum | Chryseobacterium_indologenes | 0.3588 | 1 | 11.87 | 12.49 | 328 | 354 |
|  |  | Torque_teno_virus | 1.33 | 1 | 3.5 | / | 1 | / |
|  |  | Human_alphaherpesvirus_1 | 0.0336 | 1 | 0.04 | / | 1 | / |
| 518 | Sputum | Enterococcus_faecalis | 0.0124 | 1 | 0.05 | 71.12 | 0 | 11458 |
|  |  | Serratia_rubidaea | 0 | 0 | 0 | 0 | 0 | 0 |
|  |  | Stenotrophomonas_maltophilia | 0 | 0 | 0 | 0 | 0 | 0 |
|  |  | Candida_albicans | 0 | 0 | 0 | 0 | 0 | 0 |
|  |  | Human_herpesvirus_4 | 0.0712 | 1 | 2.92 | / | 0 | / |
|  |  | Leishmania_mexicana | 0 | 0 | 0 | 0 | 0 | 0 |
|  |  | Clonorchis_sinensis | 0 | 0 | 0 | 0 | 0 | 0 |
| 529 | Sputum | Enterobacter_cloacae | 0 | 0 | 0 | 0 | 0 | 0 |
|  |  | Salmonella_enterica_serotype_Typhi | 0 | 0 | 0 | 0 | 0 | 0 |
|  |  | Salmonella_bongori | 0 | 0 | 0 | 0 | 0 | 0 |
|  |  | Human_herpesvirus_4 | 0 | 0 | 0 | 0 | 0 | 0 |
|  |  | Human_betaherpesvirus_5 | 0 | 0 | 0 | 0 | 0 | 0 |
|  |  | Torque_teno_virus | 0 | 0 | 0 | 0 | 0 | 0 |
|  |  | Human_papillomavirus_type_36 | 0 | 0 | 0 | 0 | 0 | 0 |
|  |  | Human_papillomavirus_98 | 0 | 0 | 0 | 0 | 0 | 0 |
|  |  | Human_betaherpesvirus_7 | 0 | 0 | 0 | 0 | 0 | 0 |
|  |  | Human_betaherpesvirus_6B | 0 | 0 | 0 | 0 | 0 | 0 |
|  |  |  |  |  |  |  |  |  |
| 532 | CSF | Pseudomonas_aeruginosa | 0.0074 | 1 | 0.25 | 0.71 | 1 | 4 |
|  |  | Aspergillus_fumigatus | 0 | 0 | 0 | 0 | 0 | 0 |
|  |  | Aspergillus_niger | 0 | 0 | 0 | 0 | 0 | 0 |
|  |  | Aspergillus_terreus | 0 | 0 | 0 | 0 | 0 | 0 |
|  |  | Candida_parapsilosis | 0 | 0 | 0 | 0 | 0 | 0 |
|  |  | Candida_tropicalis | 0 | 0 | 0 | 0 | 0 | 0 |
|  |  | Human_papillomavirus_167 | 0 | 0 | 0 | 0 | 0 | 0 |
|  |  | Human_alphaherpesvirus_2 | 0 | 0 | 0 | 0 | 0 | 0 |
|  |  | Human_papillomavirus_120 | 0 | 0 | 0 | 0 | 0 | 0 |
|  |  | Human_papillomavirus_98 | 0 | 0 | 0 | 0 | 0 | 0 |
| 563 | Sputum | Human_betaherpesvirus_7 | 0 | 0 | 0 | 0 | 0 | 0 |
| 584 | Hydrothorax | Pseudomonas_aeruginosa | 0.0073 | 1.02 | 0.26 | 0.54 | 4 | 6 |
|  |  | Human_betaherpesvirus_5 | 0 | 0 | 0 | 0 | 0 | 0 |
| 598 | Bile | Torque_teno_virus_16 | 0 | 0 | 0 | 0 | 0 | 0 |
| 615 | Pus | Candida_albicans | 0.0001 | 1 | 1.28 | 15 | 0 | 5 |
|  |  | Human_herpesvirus_4 | 0 | 0 | 0 | 0 | 0 | 0 |
|  |  | Human_betaherpesvirus_5 | 0 | 0 | 0 | 0 | 0 | 0 |
| 618 | Drainage | Bacteroides_plebeius | 0.0046 | 1 | 1.04 | 15.95 | 4 | 88 |
|  |  | Parabacteroides_merdae | 0 | 0 | 0 | 0 | 0 | 0 |
|  |  | Human_betaherpesvirus_6B | 0 | 0 | 0 | 0 | 0 | 0 |
|  |  |  |  |  |  |  |  |  |
| 625 | BALF | Acinetobacter_baumannii | 1.29 | 1.01 | 21.22 | 23.19 | 658 | 982 |
|  |  | Klebsiella_pneumoniae | 0.4464 | 1.01 | 6.99 | 9.48 | 212 | 597 |
|  |  | Aspergillus_fumigatus | 0.0002 | 1 | 3.31 | 6.62 | 1 | 1 |
|  |  | Torque_teno_virus | 7.19 | 4.09 | 91.61 | / | 13 | / |
|  |  | Torque_teno_virus_14 | 1.28 | 1 | 1.57 | / | 1 | / |
| 626 | Sputum | Streptococcus_pneumoniae | 0.0023 | 1 | 0.1 | 2.15 | 1 | 19 |
|  |  | Klebsiella_pneumoniae | 0.0008 | 1 | 0.02 | 0.07 | 0 | 1 |
|  |  | Pneumocystis_jirovecii | 0 | 0 | 0 | 0 | 0 | 0 |
|  |  | Human_betaherpesvirus_7 | 0 | 0 | 0 | 0 | 0 | 0 |
|  |  | Human_alphaherpesvirus_1 | 0 | 0 | 0 | 0 | 0 | 0 |
|  |  | Human_betaherpesvirus_6B | 0 | 0 | 0 | 0 | 0 | 0 |
| 627 | Bile | Enterococcus_faecalis | 0 | 0 | 0 | 0 | 0 | 0 |
|  |  | Pyramidobacter_piscolens | 0 | 0 | 0 | 0 | 0 | 0 |
|  |  | Mogibacterium_timidum | 0 | 0 | 0 | 0 | 0 | 0 |
|  |  | Streptobacillus_moniliformis | 0 | 0 | 0 | 0 | 0 | 0 |
|  |  | Bilophila_wadsworthia | 0 | 0 | 0 | 0 | 0 | 0 |
|  |  | Salmonella_enterica_serotype_Typhi | 0 | 0 | 0 | 0 | 0 | 0 |
|  |  | Enterobacter_cloacae | 0 | 0 | 0 | 0 | 0 | 0 |
|  |  | Escherichia_coli | 0 | 0 | 0 | 0 | 0 | 0 |
|  |  | Candida_parapsilosis | 0 | 0 | 0 | 0 | 0 | 0 |
|  |  | Candida_dubliniensis | 0 | 0 | 0 | 0 | 0 | 0 |
|  |  | Candida_tropicalis | 0 | 0 | 0 | 0 | 0 | 0 |
|  |  | Candida_albicans | 0 | 0 | 0 | 0 | 0 | 0 |
|  |  | Human_betaherpesvirus_5 | 0 | 0 | 0 | 0 | 0 | 0 |
| 627 | Sputum | Stenotrophomonas_maltophilia | 0 | 0 | 0 | 0 | 0 | 0 |
|  |  | Human_betaherpesvirus_7 | 0 | 0 | 0 | 0 | 0 | 0 |
|  |  | Human_betaherpesvirus_5 | 0 | 0 | 0 | 0 | 0 | 0 |
| 629 | Ascitic fluid | Acinetobacter_baumannii | 0 | 0 | 0 | 0 | 0 | 0 |
| 711 | Sputum | Aspergillus_fumigatus | 0 | 0 | 0 | 0 | 0 | 0 |
| 714 | Sputum | Acinetobacter_baumannii | 0.0012 | 1 | 0.21 | 1.39 | 1 | 6 |
|  |  | Stenotrophomonas_maltophilia | 0.0021 | 1 | 0.37 | 0.37 | 1 | 1 |
|  |  | Human_herpesvirus_7 | 0 | 0 | 0 | 0 | 0 | 0 |
| 741 | Sputum | Staphylococcus_epidermidis | 0.0706 | 1 | 3.8 | 4.02 | 34 | 37 |
|  |  | Human_herpesvirus_4 | 0 | 0 | 0 | 0 | 0 | 0 |
| 743 | CSF | Mycobacterium_tuberculosis_complex_group | 0 | 0 | 0 | 0 | 0 | 0 |
| 747 | Sputum | Veillonella_parvula | 0.0094 | 1 | 0.36 | 0.45 | 3 | 4 |
|  |  | Enterococcus_faecalis | 0.0179 | 1 | 0.7 | 1.19 | 3 | 16 |
|  |  | Candida_albicans | 0 | 0 | 0 | 0 | 0 | 0 |
| 749 | BALF | Pneumocystis_jirovecii | 0 | 0 | 0 | 0 | 0 | 0 |
| 751 | BALF | Corynebacterium_striatum | 0.0034 | 1 | 0.16 | 1.22 | 2 | 11 |
|  |  | Enterococcus_faecium | 0.0034 | 1 | 0.16 | 0.32 | 2 | 4 |
| 770 | BALF | Human_herpesvirus_7 | 0.0327 | 1 | 0.05 | / | 1 | / |
|  |  | Torque_teno_mini_virus_7 | 0 | 0 | 0 | 0 | 0 | 0 |
| 779 | BALF | Haemophilus_parainfluenzae | 0.0207 | 1 | 0.5 | 0.56 | 4 | 4 |
|  |  | Pseudomonas_aeruginosa | 0.0204 | 1 | 0.48 | 0.77 | 24 | 25 |
|  |  | Human_herpesvirus_1 | 0 | 0 | 0 | 0 | 0 | 0 |
| 814 | Sputum | Acinetobacter_baumannii | 0.0037 | 1 | 0.11 | 0.79 | 1 | 11 |
| 816 | BALF | Stenotrophomonas_maltophilia | 0.0022 | 1 | 0.06 | 0.06 | 0 | 0 |
| 821 | BALF | Acinetobacter_baumannii | 0 | 0 | 0 | 0 | 0 | 0 |
|  |  | Torque_teno_virus_8 | 0 | 0 | 0 | 0 | 0 | 0 |
| 828 | Pus | Citrobacter_freundii | 0.001 | 1 | 0.02 | 0.1 | 1 | 3 |
|  |  | Kluyvera_georgiana | 0 | 0 | 0 | 0 | 0 | 0 |
|  |  | Escherichia_coli | 0.0213 | 1 | 0.53 | 0.69 | 1 | 2 |
|  |  | Aeromonas_veronii | 0.0029 | 1 | 0.08 | 0.15 | 2 | 3 |
|  |  | Candida_albicans | 0 | 0 | 0 | 0 | 0 | 0 |
| 839 | Sputum | Human_herpesvirus_1 | 0 | 0 | 0 | 0 | 0 | 0 |
| 842 | Sputum | Human_herpesvirus_7 | 0 | 0 | 0 | 0 | 0 | 0 |
| 845 | CSF | Shigella_dysenteriae | 0 | 0 | 0 | 0 | 0 | 0 |
|  |  | Shigella_boydii | 0 | 0 | 0 | 0 | 0 | 0 |
| 854 | BALF | Mycoplasma_pneumoniae | 0.0224 | 1 | 0.5 | 0.5 | 3 | 3 |
|  |  | Torque_teno_virus_3 | 0 | 0 | 0 | 0 | 0 | 0 |
| 857 | BALF | Mycobacterium | / | / | / | 0.06 | / | 0 |
| 871 | BALF | Torque_teno_virus_27 | 0 | 0 | 0 | 0 | 0 | 0 |
| 887 | BALF | Pseudomonas_aeruginosa | 0.0007 | 1 | 6.75 | 6.75 | 1 | 1 |
|  |  | Stenotrophomonas_maltophilia | 0 | 0 | 0 | 0 | 0 | 0 |
| 888 | Pus | Enterobacter_cloacae | 0.0092 | 1 | 0.61 | 2.18 | 2 | 21 |
|  |  | Hafnia_paralvei | 0.002 | 1 | 0.13 | 0.4001 | 2 | 2 |
|  |  | Escherichia_coli | 0.0544 | 1 | 3.6 | 5.09 | 4 | 7 |
|  |  | Citrobacter_koseri | 0 | 0 | 0 | 0 | 0 | 0 |
|  |  |  |  |  |  |  |  |  |
| 890 | Sputum | Haemophilus_parainfluenzae | 0 | 0 | 0 | 0 | 0 | 0 |
|  |  | Neisseria_flavescens | 0.0043 | 1 | 2.21 | 4.71 | 0 | 3 |
|  |  | Neisseria_subflava | 0.0022 | 1 | 1.13 | 4.71 | 0 | 3 |
|  |  | Neosartorya_fischeri | 0 | 0 | 0 | 0 | 0 | 0 |
|  |  | Torque_teno_virus_10 | 1.33 | 1 | 6.41 | / | 0 | / |
|  |  | Torque_teno_virus_15 | 0 | 0 | 0 | 0 | 0 | 0 |
|  |  | Human_herpesvirus_1 | 0 | 0 | 0 | 0 | 0 | 0 |
| 900 | BALF | Staphylococcus_haemolyticus | 0.013 | 1 | 2.81 | 3.66 | 6 | 9 |
|  |  | Elizabethkingia_anophelis | 0 | 0 | 0 | 0 | 0 | 0 |
|  |  | Human_herpesvirus_7 | 0 | 0 | 0 | 0 | 0 | 0 |
|  |  | Human_herpesvirus_6B | 0 | 0 | 0 | 0 | 0 | 0 |
| 903 | Sputum | Acinetobacter_baumannii | 0.0012 | 1 | 0.1 | 2.26 | 0 | 12 |
|  |  | Haemophilus_parainfluenzae | 0.0024 | 1 | 0.19 | 0.19 | 1 | 1 |
|  |  | Torque_teno_virus_28 | 0 | 0 | 0 | 0 | 0 | 0 |
| 920 | Tissue | Peptostreptococcus_stomatis | 0 | 0 | 0 | 0 | 0 | 0 |
|  |  | Prevotella_timonensis | 0.0016 | 1 | 0.4 | 3.48 | 0 | 5 |
| 924 | Sputum | Haemophilus_parainfluenzae | 0 | 0 | 0 | 0 | 0 | 0 |
|  |  | Pseudomonas_aeruginosa | 0.0007 | 1 | 0.07 | 1.17 | 1 | 6 |
|  |  | Citrobacter_koseri | 0 | 0 | 0 | 0 | 0 | 0 |
|  |  | Raoultella_ornithinolytica | 0 | 0 | 0 | 0 | 0 | 0 |
|  |  |  |  |  |  |  |  |  |
| 934 | Sputum | Stenotrophomonas_maltophilia | 0.0022 | 1 | 0.02 | 0.02 | 2 | 2 |
|  |  | Acinetobacter_baumannii | 0.0037 | 1 | 0.03 | 0.25 | 0 | 13 |
|  |  | Klebsiella_pneumoniae | 0.0018 | 1 | 0.01 | 0.04 | 1 | 3 |
|  |  | Human_herpesvirus_1 | 0 | 0 | 0 | 0 | 0 | 0 |
|  |  | Human_herpesvirus_7 | 0 | 0 | 0 | 0 | 0 | 0 |
|  |  | Mycobacterium_tuberculosis_complex_group | 0 | 0 | 0 | 0 | 0 | 0 |
| 946 | Sputum | Haemophilus_parainfluenzae | 0 | 0 | 0 | 0 | 0 | 0 |
|  |  | Streptococcus_pneumoniae | 0 | 0 | 0 | 0 | 0 | 0 |
|  |  | Klebsiella_pneumoniae | 0 | 0 | 0 | 0 | 0 | 0 |
|  |  | Mycobacterium_tuberculosis_complex_group | 0 | 0 | 0 | 0 | 0 | 0 |
|  |  | Human_herpesvirus_7 | 0 | 0 | 0 | 0 | 0 | 0 |
|  |  | Hepatitis_B_virus | 0 | 0 | 0 | 0 | 0 | 0 |
|  |  | Torque_teno_virus_3 | 0 | 0 | 0 | 0 | 0 | 0 |
| 984 | BALF | Acinetobacter_baumannii | 0.0037 | 1 | 1.25 | 3.13 | 2 | 5 |
|  |  | Pseudomonas_aeruginosa | 0 | 0 | 0 | 0 | 0 | 0 |
|  |  | Human_herpesvirus_4 | 0.0579 | 1 | 1.66 | / | 1 | / |
| 1012 | Bile | Corynebacterium | / | / | / | 0.47 | / | 2 |
|  |  | Candida_tropicalis | 0 | 0 | 0 | 0 | 0 | 0 |
|  |  | Human_parvovirus_B19 | 0 | 0 | 0 | 0 | 0 | 0 |
| 1012 | Sputum | Staphylococcus_epidermidis | 0.0076 | 1 | 0.51 | 1.13 | 3 | 7 |
|  |  | Enterococcus_faecalis | 0 | 0 | 0 | 0 | 0 | 0 |
|  |  |  |  |  |  |  |  |  |
| 1026 | Bile | Enterococcus_faecalis | 0.0031 | 1 | 0.07 | 0.07 | 2 | 2 |
|  |  | Escherichia_coli | 0.0019 | 1 | 0.07 | 0.09 | 1 | 1 |
|  |  | Enterobacter_cloacae | 0 | 0 | 0 | 0 | 0 | 0 |
|  |  | Candida_tropicalis | 0.0003 | 1 | 1.33 | 1.33 | 1 | 1 |
| 1026 | Sputum | Acinetobacter_baumannii | 0.0438 | 1.01 | 1.18 | 8.81 | 26 | 234 |
|  |  | Stenotrophomonas_maltophilia | 0.0019 | 1 | 0.09 | 0.41 | 2 | 6 |
|  |  | Escherichia_coli | 0.0019 | 1 | 0.07 | 0.09 | 1 | 1 |
|  |  | Human_alphaherpesvirus_1 | 0 | 0 | 0 | 0 | 0 | 0 |
